# *Candida albicans* metabolic adaptation gene *SFU1* regulates dual-species biofilm with *Streptococcus mutans*

**DOI:** 10.3389/fcimb.2026.1795742

**Published:** 2026-03-09

**Authors:** Qian Jiang, Jiang Lin

**Affiliations:** Department of Stomatology, Beijing Tongren Hospital, Capital Medical University, Beijing, China

**Keywords:** biofilm, Candida albicans, dental caries, SFU1, Streptococcus mutans

## Abstract

**Objective:**

To investigate the role of the iron-sulfur cluster assembly factor *SFU1* in the virulence-related traits of *Candida albicans*, particularly its function within the cariogenic cross-kingdom biofilm formed with *Streptococcus mutans*.

**Methods:**

The *SFU1* deletion and complemented strains were constructed. Their effects on growth, acid production, morphogenesis, metabolic activity, ROS accumulation, and biofilm formation of *C. albicans* were evaluated. The roles of *SFU1* in the development, architecture, and spatial distribution of the *C. albicans-S. mutans* dual-species biofilm were further analyzed. The cariogenic metabolite profile and matrix synthesis were assessed by measuring lactic acid production, lactate dehydrogenase activity, extracellular polysaccharide content, and expression levels of related genes.

**Results:**

The *SFU1* deletion strain exhibited inhibited hyphal formation, reduced metabolic activity, elevated intracellular ROS levels, impaired biofilm formation, and downregulated expression of hyphal and adhesion-related genes (*ALS3, EFG1, UME6*). In the cross-kingdom biofilm, the *sfu1/sfu1* mutant failed to form hyphal networks, resulting in loose biofilm architecture, reduced biomass, and poor integration of *S. mutans*. Furthermore, the dual-species biofilm showed significantly decreased lactic acid and EPS production. Co-cultured *S. mutans* exhibited downregulated expression of EPS synthesis genes (*gtfB/C*) and upregulated expression of EPS degradation genes (*dexA/B*).

**Conclusion:**

*SFU1* modulates hyphal development, redox homeostasis, and biofilm formation in *C. albicans*, thereby profoundly affecting its pathogenic synergy with *S. mutans*. *SFU1* deletion leads to disrupted architecture and attenuated cariogenic virulence of the dual-species biofilm. This study reveals the potential value of targeting fundamental metabolic pathways in *C. albicans* to interfere with the cariogenicity of cross-kingdom biofilms, and provides a novel perspective for the prevention and therapy of dental caries.

## Introduction

Dental caries remains one of the most prevalent chronic infectious diseases worldwide, affecting up to approximately 2.4 billion people, causing serious impacts on people’s oral health and socio-economic burden (Peres et al., 2019; Wen et al., 2022). While the classic “specific plaque hypothesis” emphasizes the pivotal role of acidogenic bacteria such as *Streptococcus mutans*, the emerging “ecological plaque hypothesis” underscores that caries development results from a polymicrobial imbalance within the oral community, wherein cross-kingdom interactions between fungi and bacteria play a crucial role (Baker et al., 2024; Sedghi et al., 2021). *Candida albicans*, a common opportunistic fungal pathogen, is frequently detected at high levels in dental plaque biofilms from early childhood caries and rampant caries, with its abundance positively correlating with caries severity (Kashyap et al., 2024; Lu et al., 2023; Francis et al., 2025; Man et al., 2025).

Accumulating evidence reveals that *C. albicans* and *S. mutans* do not merely coexist but form a highly organized symbiotic consortium within biofilms. This synergistic relationship markedly enhances the cariogenic potential of the biofilm: *C. albicans* provides structural scaffolding through hyphal formation and adhesins, facilitating bacterial adhesion and biofilm accumulation, while its ability to metabolize bacterial-derived lactate mitigates localized acid stress, thereby promoting a continuously acidic microenvironment conducive to enamel demineralization. Moreover, these cross-kingdom biofilms exhibit enhanced resistance to antimicrobial agents and host immune clearance (Du et al., 2021; Sztajer et al., 2014; Jin et al., 2024; Xiao et al., 2022). Hence, elucidating the molecular mechanisms that enable *C. albicans* to thrive, persist, and functionally collaborate within cariogenic symbiotic biofilms is essential for advancing our understanding of caries pathogenesis and developing novel therapeutic strategies.

Research on the cariogenic role of *C. albicans* has largely focused on classical virulence factors such as adhesins, hyphal morphogenesis, and acid production. However, in the dynamic, competitive, and often nutrient-limited oral biofilm microenvironment, the metabolic adaptability and stress response capacity constitute the fundamental basis for the long-term survival and pathogenicity (Lamont et al., 2018). Iron-sulfur (Fe-S) clusters, ancient and essential protein cofactors, are involved in core cellular processes including mitochondrial respiration, amino acid biosynthesis, antioxidant defense, and DNA repair (Lill and Freibert, 2020; Braymer and Lill, 2017). The *SFU1* gene encodes the central cysteine desulfurase required for Fe-S cluster biogenesis in *C. albicans*, serving as a metabolic hub that integrates energy production and stress adaptation. Deletion of *SFU1* leads to severe growth defects, particularly in mitochondrial respiration and non-fermentable carbon source utilization, along with heightened sensitivity to various environmental stresses such as oxidative and cell wall stress (Andreieva et al., 2020; Zheng et al., 2025; Garg et al., 2025). These phenotypes suggest that the metabolic and stress-responsive capacities governed by *SFU1* may represent a core fitness determinant for *C. albicans* within the competitive oral biofilm ecosystem. Nevertheless, it remains completely unknown whether and how *SFU1* modulates the role of *C. albicans* in cross-kingdom interactions with *S. mutans*, thereby influencing the structure and cariogenicity of the symbiotic biofilm.

Based on the above, we hypothesize that the *SFU1* gene, by maintaining Fe-S cluster homeostasis and supporting metabolic adaptation in *C. albicans*, critically regulates its ability to colonize, promote biofilm maturation, tolerate acid stress, and ultimately enhance the cariogenic potential of *C. albicans-S. mutans* biofilm. This study aims to systematically test this hypothesis through phenotypic, metabolic, and molecular analyses of the wild-type (WT) and *sfu1/sfu1* mutant strains within dual-species biofilm models. Our work will not only provide a novel “metabolic adaptation” perspective on the pathogenesis of dental caries but may also identify *SFU1* as a potential target for ecological interventions aimed at modulating polymicrobial biofilm communities and developing innovative anti-caries strategies.

## Materials and methods

### Strains and culture conditions

The *C. albicans* strains used in this study are listed in **Table 1**. The *SFU1* knockout mutant was constructed by fusion PCR strategy (Noble and Johnson, 2005). Plasmids pSN52 and pSN40 were used for amplification of HIS1 and LEU2 markers, respectively. *C. albicans* genomic DNA was used for amplification of the 5’- and 3’- flanking fragments of the corresponding genes. The HIS1 and LEU2 markers flanked by *SFU1* 5’- and 3’- fragments were amplified with fusion PCR. The PCR products of the HIS1 and LEU2 markers were sequentially transformed into *C. albicans* WT, generating the *SFU1* deletion mutant. The *SFU1* reconstituted strain was constructed using pNIM1 plasmid. Fragments of the 3′-untranslated region (UTR) and that containing the open reading frame (ORF) plus 5′-UTR of *SFU1* were amplified by PCR from the genomic DNA of SN250. The PCR products were digested with XhoI/BglII and SalI/BamHI respectively, and subcloned into the plasmid pNIM1 (Park and Morschhauser, 2005). The *SFU1* reconstituted strain was constructed by transforming the *sfu1/sfu1* mutant with the SalI-digested pNIM1-SFU1p-SFU1 plasmid. Primers used in this study are listed in **Supplementary Table 1**. The *S. mutans* wild type strain UA159 were commercially obtained from the American Type Culture Collection.

**Table 1:** *Candida albicans* strains used in this study.

Strain	Parent	Genotype	Source
SN250	CAI4	*ura3::imm434::URA3-IRO1/ura3::imm434 arg4::hisG/arg4::hisG his1::hisG/his1::hisG leu2::hisG::CdHIS1/leu2::hisG::CmLEU2*	(Nobile and Mitchell, 2005)
*sfu1/sfu1*	SN250	As SN250, but *sfu1::LEU2/sfu1::HIS1*	This study
*sfu1/sfu1* + *SFU1*	*sfu1/sfu1*	As *sfu1/sfu1*, but SFU1p-SFU1	This study

YPD medium (20 g/L glucose, 20 g/L peptone, and 10 g/L yeast extract) was used for routine culture of *C. albicans*. Brain-heart infusion (BHI) medium was used for routine culture of *S. mutans*. Spider medium (10 g/L nutrient broth, 10 g/L mannitol, and 2.6207 g/L K_2_HPO_4_•3H_2_O) was used for hyphal development of *C. albicans*. YNBB medium (0.67% YNB, 75 mM Na_2_HPO_4_, 75 mM NaH_2_PO_4_, 2.5 mM N-acetylglucosamine, 0.2% casamino acids, and 0.5% sucrose) was used for co-culture of *S. mutans* and *C. albicans*. Solid medium was supplemented with 2% agar.

*C. albicans* was incubated at 30 °C (for routine culture) or 37 °C (for hyphal induction) aerobically. *S. mutans* was incubated at 37 °C anaerobically (90% N_2_, 5% H_2_, 5% CO_2_). For dual-species biofilm, *C. albicans* and *S. mutans* were co-incubated at 37 °C aerobically.

### Growth curve and pH value

*C. albicans* cells were incubated in YPD liquid medium overnight to stationary phase, and then transferred to fresh YPD with an initial concentration of 5000 cells/mL. The cells were incubated at 30 °C with shaking at 200 rpm. The cell densities and pH values of supernatant were detected at different time points. Three independent repeats were performed.

### Colony and cell morphology of *C. albicans*

Cell suspension containing approximately 100 C*. albicans* cells was spread onto YPD or Spider solid medium and incubated at 37 °C for 3 days. Colony morphology was observed using a stereo microscope (SZX16, Olympus, Japan), and cells were then collected for cellular morphology observation under an upright microscope (DM2500, Leica, Germany).

### ROS generation

ROS levels were measured using a ROS assay kit (Beyotime Biotech, China). *C. albicans* cells (1 × 10^6^ cells/mL) were incubated for 2 h at 30 °C with shaking, and stained with 10 µM of 2′,7′-dichlorofluorescein diacetate (DCFH-DA) for 30 min at 37 °C in the dark. Cells were observed using an inverted fluorescence microscope (Observer Z1, Zeiss, Germany). Intracellular ROS levels were visually reflected by the green fluorescence emitted from the DCFH-DA reaction with ROS. The fluorescence intensity was measured with the microplate reader at excitation and emission wavelengths of 488 and 525 nm, respectively.

### Metabolic activity measurement by XTT assay

The metabolic activity of *C. albicans* cells was determined using a colorimetric 2,3-bis (2-methoxy- 4-nitro-5-sulfophenyl)-2 H-tetrazolium-5-carboxanilide (XTT) assay (Gulati et al., 2018). Solutions containing 0.5 mg/mL XTT and 0.32 mg/mL PMS were freshly prepared by dissolving XTT powder and PMS powder in PBS and water, respectively. The solutions were filter-sterilized (0.22 μm pore size filter), mixed at a 9:1 XTT: PMS ratio, and protected from light. *C. albicans* cells were suspended in XTT: PMS solution, adjusted to 1 × 10^7^ cells/mL, and transferred to a 96-well microtiter plate, 100 μL for each well. The plate was incubated in the dark for 30 min at 37 °C, and the optical density was measured at 492 nm using a microplate reader.

### Biofilm biomass assay by crystal violet staining

For biofilm formation, the initial density of *C. albicans* was adjusted to 1 × 10^4^ cells/mL and *S. mutans* to 1 × 10^6^ CFU/mL. After being incubated in 96-well microtiter plate for 24 h, the biofilm was gently washed with PBS and stained with 0.1% crystal violet for 15 min. Then, the stained biofilm was washed again with PBS and the crystal violet was solubilize with 33% glacial acetic acid. The optical density was measured with the microplate reader at 570 nm.

### Gene expression analysis using qRT-PCR

*C. albicans* cells were incubated overnight at 30 °C with shaking and harvested at mid-exponential phase by centrifugation. Biofilms were harvested by scraping after incubation in 6-wellmicrotiter plate with YNBB for 24 h. Total RNA was extracted using TRIzol reagent (Thermo Fisher Scientific, USA) according to manufacturer’s instructions, and cDNA was prepared using RevertAid Reverse Transcriptase (Thermo Fisher Scientific, USA). Quantitative reverse real-time PCR (qRT-PCR) was performed in a Bio-Rad CFX96 real-time PCR detection system using SYBR Green qPCR mix (TOYOBO, Japan). The expression levels of each experimental sample were normalized to those of ACT1 (for *C. albicans*) or gyrA (for *S. mutans*). Primers used in this analysis are listed in **Supplementary Table 1**.

### Biofilm morphology under SEM

The *C. albicans-S. mutans* biofilm after 24 h incubation in YNBB were fixed with 2.5% (v/v) glutaraldehyde for 2 h at 4 °C. The fixed samples were washed twice with phosphate buffered saline (PBS), dehydrated in a series of ethanol solutions (50, 75, and 90% for 10 min and then absolute alcohol for 10 min twice), and subsequently treated with a series of tert-Butanol solutions (50, 75, and 90% for 10 min and then absolute tert-Butanol for 10 min twice). The samples were freeze-dried, coated with a thin layer of gold-palladium, and observed under a scanning electron microscope (TM-3000, Hitachi, Japan).

### Distribution of *C. albicans* and *S. mutans*

Fluorescence *in situ* hybridization (FISH) was performed to label *C. albicans* and *S. mutans* with species-specific probes. *C. albicans* and *S. mutans* were co-incubated in confocal dishes at 37 °C for 24 h in the dark for biofilm formation. After washing with PBS, 4% paraformaldehyde solution was added to fix the biofilms at 4 °C for 10 h. The fixed biofilms were washed and dried at 46 °C for 15 min, incubated in lysis buffer (0.1 M Tris-HCl, 50 mM EDTA, 30 g/L lysozyme) at 37 °C for 20 min, dehydrated in a series of ethanol solutions (50, 80, and 96% for 3 min respectively), and dried at 46 °C for 10 min. The FISH probes were dissolved and mixed with hybridization buffer (20 mM Tris-HCl, 0.9 M NaCl, 20% formamide, 0.01% SDS), and then added to the samples. After incubated at 46 °C for 90 min in the dark, the samples were treated with washing buffer (20 mM Tris-HCl, 5 mM EDTA, 215 mM NaCl, 0.01% SDS) and incubated at 48 °C for 15 min in the dark. The biofilm samples were observed by CLSM with a 20× objective lens. The 3D images were reconstructed by Application Suite X (LAS X) software (Leica, Germany), and quantitative analysis of fluorescence was performed using ImageJ.

### EPS production and composition assessment by anthrone method

Biofilms were harvested and vortexed in PBS buffer. The supernatant and sediment were harvested respectively after centrifugation (4000 rpm for 15 min at 4 °C). The supernatant containing water-soluble polysaccharides (WSG) was filtered through a 0.22 µm filter. Then, 20% trichloroacetic acid was added and the mixture was placed at 4 °C for 2 h. The solution was centrifuged and the supernatant was collected for WSG measurement. The sediment was resuspended in 1 mL of 1 M NaOH and placed at 37 °C for 3 h with shaking. After centrifugation, the supernatant was collected for water-insoluble polysaccharides (WIG) measurement. Then, 600 μL of anthrone reagent was added to 200 μL of supernatant, and the mixtures were heated at 95 °C for 10 min. The absorbance of each sample at 620 nm was monitored on a microplate reader. The corresponding polysaccharide concentration was calculated according to the standard curve, which was prepared with a dextran standard using various concentrations.

### Biofilm structure under CLSM

To visualize EPS distribution, 1 μM Alexa Fluor 647 (Invitrogen, USA) were added into mix suspensions in confocal dishes before biofilm incubation. After incubation at 37 °C for 24 h in the dark, the microbe cells were labeled with 2.5 μM SYTO9 (Invitrogen, USA). The three-dimensional structures of the biofilms were observed under a confocal laser scanning microscope (TCS SP8, Leica, Germany).

### Statistical analysis

Three independent experiments were performed for all assays, and the quantitative results are presented as mean ± standard deviation. Statistical analyses were carried out using GraphPad Prism software (version 8.0, GraphPad, USA). After test for homogeneity of variance, one-way ANOVA and Dunnett’s *t* test were performed to compare differences between multiple groups. Differences were considered statistically significant if p values < 0.05.

## Results

### Role of *SFU1* in the growth, acid production, and morphology of *C. albicans*

**Figure 1:**
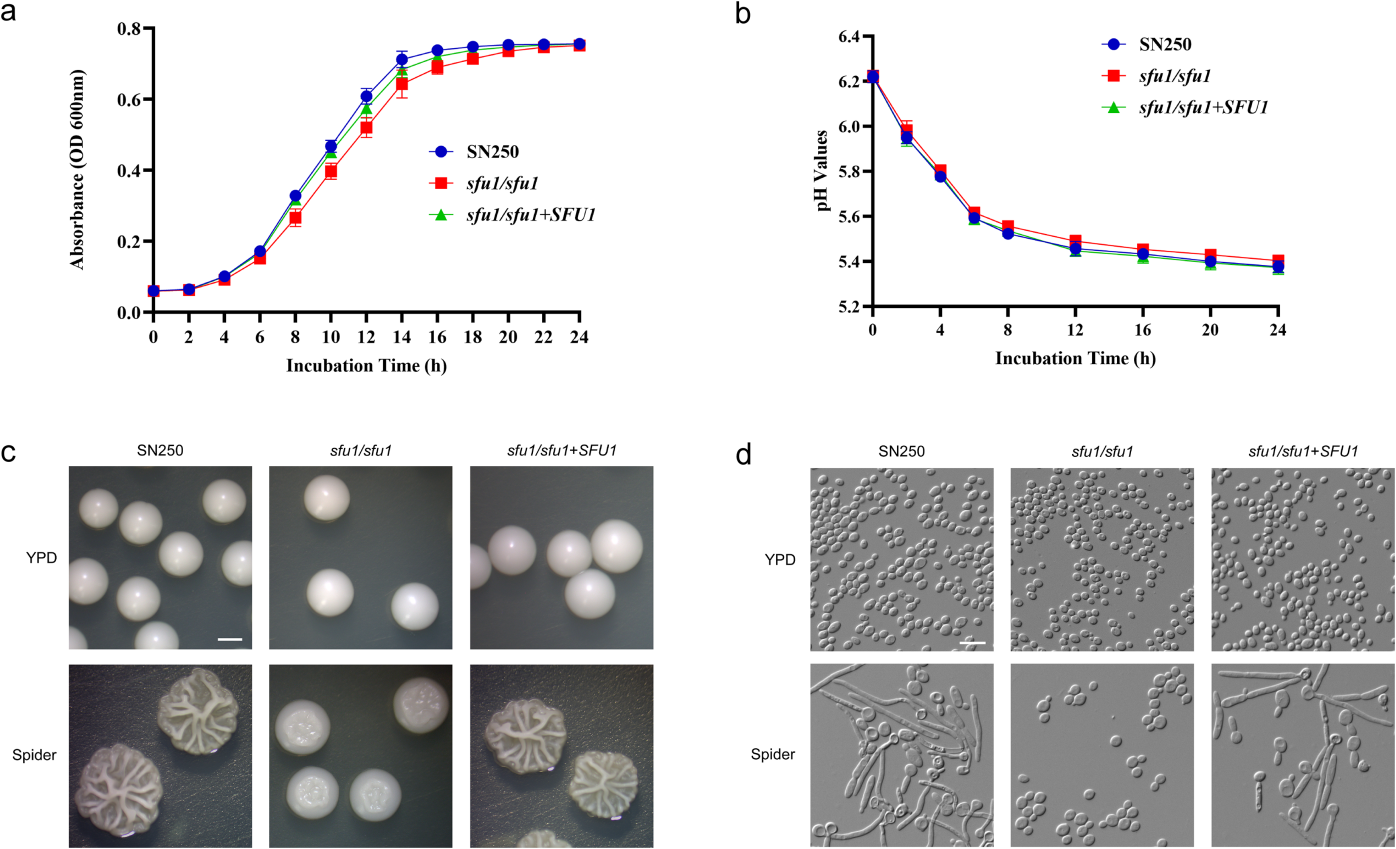
Growth, acid production, and morphology of *C. albicans*. **(A)** Growth curves. **(B)** pH values. **(C)** Colony morphology under stereo microscope. Scale bar, 1mm. **(D)** Cell morphology under upright microscope. Scale bar, 10 μm. The *C. albicans* cells at stationary phase were incubated in fresh YPD at 30 °C for 24 h detection of cell densities and pH values. The results are based on three independent experiments and represent as mean ± SD. For morphology observation, cells were incubated on YPD or Spider solid medium at 37 °C for 3 days.

To evaluate the impact of *SFU1* on the growth, acid production, and morphology of *C. albicans*, we constructed *SFU1* knockout and complemented strains. First, the 24-hour growth curves of the *C. albicans* wild-type strain SN250, as well as the *SFU1* knockout and complemented strains were measured. As illustrated in **Figure 1A**, no statistically significant difference in growth rate was observed among the three groups during the initial 0–6 hours, and they entered the stationary phase simultaneously at approximately 20 hours. Nevertheless, between 8 and 18 hours, the *sfu1/sfu1* mutant strain exhibited a slightly slower growth rate compared to the WT.

The changes in pH value were shown in **Figure 1B**. The *sfu1/sfu1* mutant exhibited similar pH changes to those of the WT and complemented strain without statistically significant difference, indicating that *SFU1* deletion does not impair glycolytic acid production of *C. albicans* under planktonic conditions.

The morphology of colony and cell were shown in **Figures 1C, D**. After 3 days incubation in YPD medium, the strains exhibited similar morphology. On Spider medium which induces hyphal formation, the *sfu1/sfu1* mutant formed smaller, smoother colonies with a markedly reduced hyphal periphery, while the *SFU1* complemented strain exhibited a phenotype similar to that of the WT, displaying a hyphal growth pattern.

### *SFU1* affected the metabolic activity, intracellular ROS, and biofilm formation of *C. albicans*

**Figure 2:**
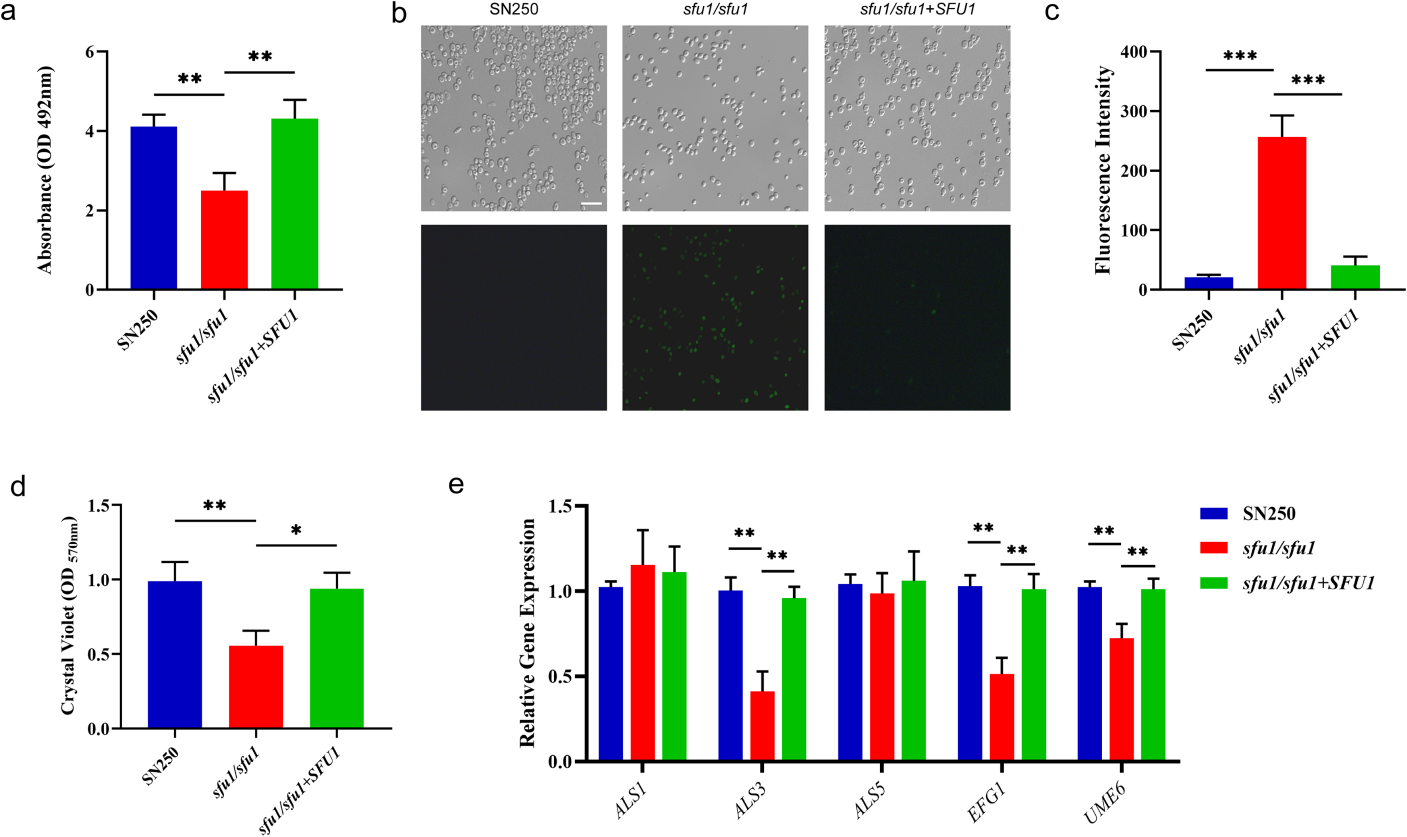
Metabolic activity, intracellular ROS, and biofilm formation of *C. albicans*. **(A)** Metabolic activity detected by XTT assay. **(B)** Intracellular ROS under inverted fluorescence microscope. Scale bar, 20 μm. **(C)** Quantitative analysis of intracellular ROS. **(D)** Biofilm biomass. **(E)** Expression of genes related to biofilm formation. The *C. albicans* cells at stationary phase were incubated in fresh YPD at 30 °C for 2 h before XTT and ROS assay. Cells were incubated in YPD at 30 °C for 24 h to form the monospecies biofilm. The results are based on three independent experiments and represent as mean ± SD. (*p<0.05, **p<0.01, ***p<0.001).

Since iron-sulfur clusters are vital for mitochondrial function, we investigated metabolic activity and redox homeostasis. The metabolic activity of the *sfu1/sfu1* mutant, as measured by the XTT assay, was significantly reduced (**Figure 2A**). Concurrently, intracellular levels of reactive oxygen species (ROS) were substantially elevated in the *sfu1/sfu1* mutant (**Figures 2B, C**), indicating that the deletion of *SFU1* can lead to ROS accumulation of *C. albicans* cells, which may result in intracellular oxidative damage and induction of apoptosis.

The biofilm biomass of *C. albicans* after 24 hours cultivation was shown in **Figure 2D**. Compared with the WT and complemented strain, the biofilm biomass of the *sfu1/sfu1* mutant decreased significantly, indicating the positive influence of *SFU1* on biofilm formation. Furthermore, the expression levels of genes related to adhesion (*ALS1, ALS3, ALS5*) and hyphal formation (*EFG1, UME6*) were detected. As shown in **Figures 2E**, the expressions of *ALS3*, *EFG1*, and *UME6* were downregulated in the *sfu1/sfu1* mutant, while the expressions of *ALS1* and *ALS5* showed no significant differences.

### *SFU1* influenced the development and architecture of *C. albicans*-*S. mutans* cross-kingdom biofilm

**Figure 3:**
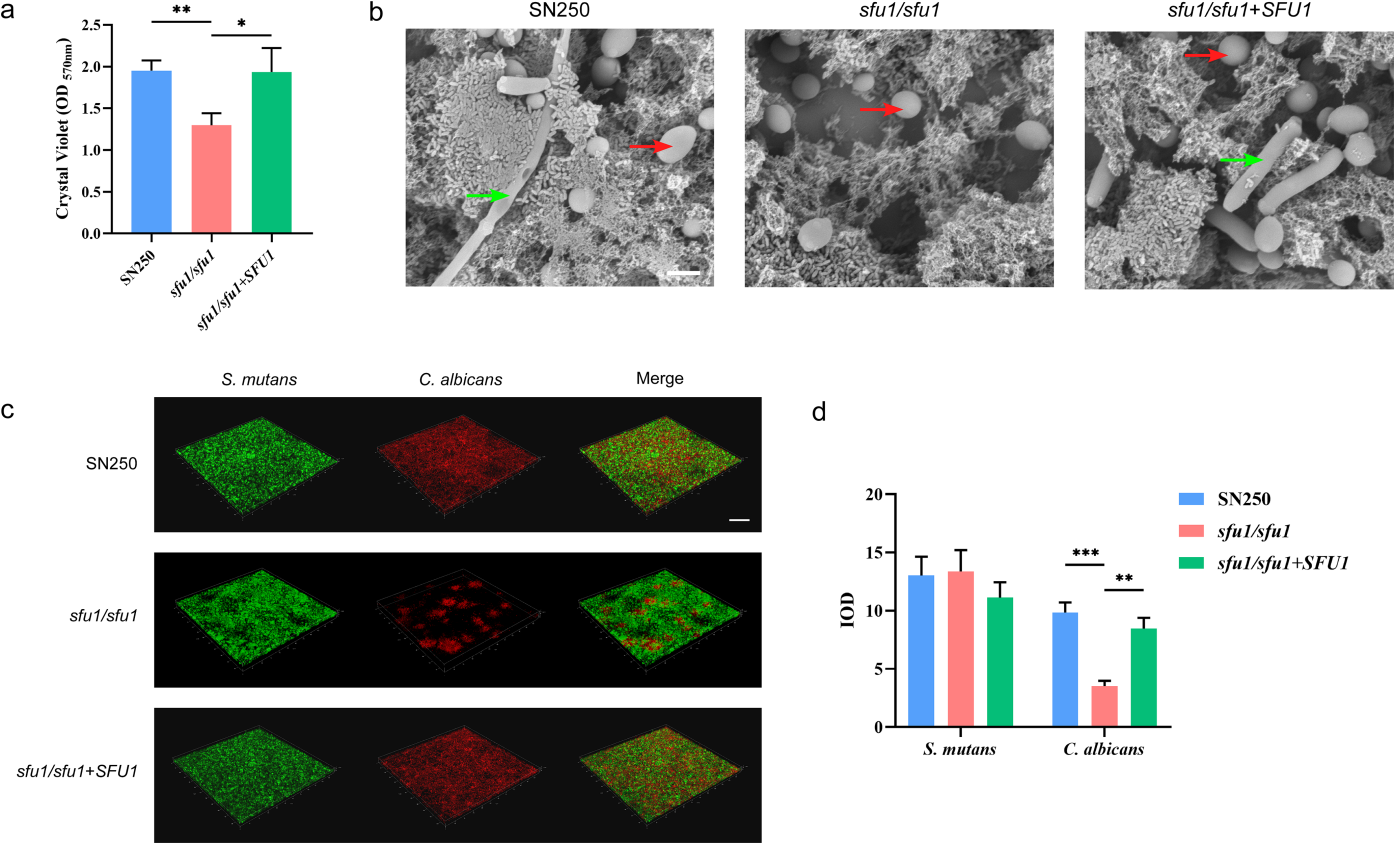
Development and architecture of *C. albicans*-*S. mutans* biofilm. **(A)** Biofilm biomass. **(B)** Biofilm morphology under SEM. The yeast state (red arrows) and hyphal state (green arrows) of *C. albicans* are presented. Scale bar, 5 μm. **(C)** Biofilm structure under CLSM. Scale bar, 100 μm. **(D)** Quantitative analysis of *C. albicans* and *S. mutans*. *C. albicans* and *S. mutans* were co-cultured in YNBB at 37 °C for 24 h to form the dual-species biofilms. The results are based on three independent experiments and represent as mean ± SD. (* p<0.05, ** p<0.01, *** p<0.001).

We then investigated whether the defects observed in monospecies biofilm would translate to the *C. albicans-S. mutans* cross-kingdom biofilm. The two species were co-cultured for 24 hours and the biofilm biomass was measured by crystal violet assay (**Figure 3A**). Compared with the WT and complemented strain, the *sfu1/sfu1* mutant showed reduced ability to form symbiotic biofilm with *S. mutans*.

Scanning electron microscopy (SEM) revealed stark architectural differences. As shown in **Figures 3B**, Biofilms formed by the WT and *SFU1* complemented strains displayed extensive hyphal networks that intimately enmeshed *S. mutans* cells, creating dense and cohesive structures with considerable thickness. In contrast, the *sfu1/sfu1* mutant primarily grew in the yeast form, resulting in loose, unstructured aggregates with poor integration of the bacterial partner.

The spatial distribution of the two species in mixed biofilm was detected by fluorescence *in situ* hybridization (FISH) and the results were shown in **Figures 3C, D**. The WT and *SFU1* complemented strains of *C. albicans* formed a uniform and stable network structure through hyphal growth, and co-adhered with *S. mutans* to establish a dense dual-species biofilm. In contrast, the *SFU1* knockout strain exhibited inhibited growth and impaired hyphal formation. The cells formed scattered, cloud-like aggregates instead of the dense network structure, and quantitative analysis revealed a significant reduction in *C. albicans* cell counts. The distribution of *S. mutans* was slightly less uniform, although its abundance showed no significant change.

### *SFU1* modulated the cariogenic metabolite profile and matrix synthesis in *C. albicans*-*S. mutans* biofilm

**Figure 4:**
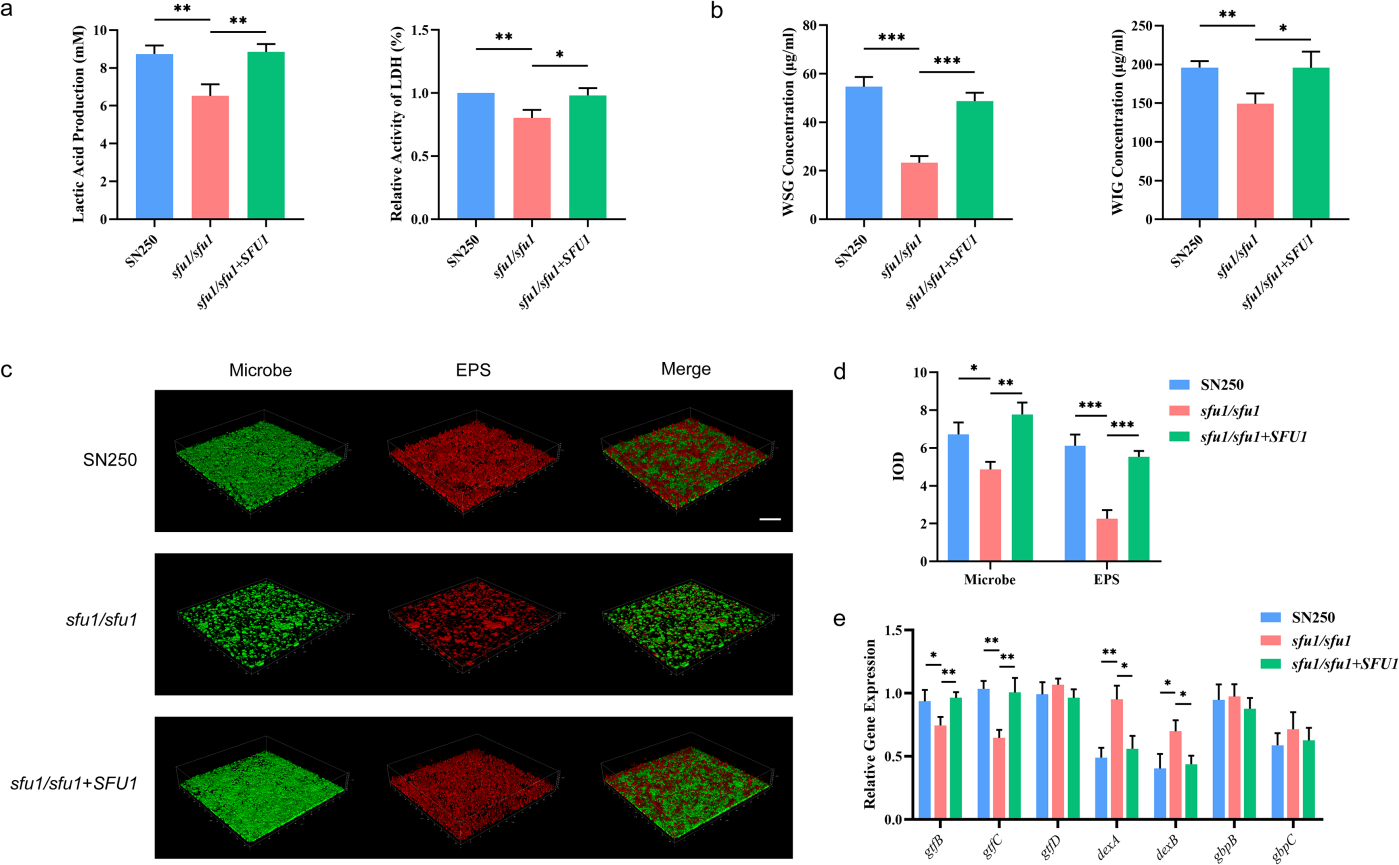
Lactic acid and EPS production of *C. albicans*-*S. mutans* biofilm. **(A)** Lactic acid production and LDH activity. **(B)** EPS generation. **(C)** Biofilm structure under CLSM. Scale bar, 100 μm. **(D)** Quantitative analysis of microbiome and EPS. **(E)** Expression of genes related to glycolysis and EPS metabolism. *C. albicans* and *S. mutans* were co-cultured in YNBB at 37 °C for 24 h to form the dual-species biofilms. The results are based on three independent experiments and represent as mean ± SD. (*p<0.05, **p<0.01, ***p<0.001).

Lactic acid produced by *S. mutans* through glycolysis is a major virulence trait of cariogenic biofilms. Therefore, we first measured the lactate production in mature biofilms formed by *C. albicans* WT and *SFU1* mutant strains in combination with *S. mutans*. As shown in **Figures 4A**, biofilms with the *sfu1/sfu1* mutant produced significantly reduced lactic acid than those with the WT and the complemented strain. The activity of lactate dehydrogenase (LDH) was also detected, and it decreased in the *sfu1/sfu1* mutant group, suggesting that *SFU1* may regulate the lactic acid production of *S. mutans* in the symbiotic biofilm by inhibiting LDH activity.

Extracellular polysaccharides (EPS) represent another key virulence determinant of cariogenic biofilms. Among these, water-insoluble glucan (WIG) promotes microbial aggregation and contributes to the structural integrity and adhesion of the biofilm matrix, while water-soluble glucan (WSG) provides adhesion sites and an energy source for microorganisms. We measured the production and composition of EPS through anthrone method and the results were demonstrated in **Figure 4B**. Compared with the WT and the complemented strain, the *sfu1/sfu1* mutant group showed significantly decreased production of both WSG and WIG.

Confocal laser scanning microscopy (CLSM) visually confirmed the paucity of both microbial biomass and EPS matrix after *SFU1* deletion (**Figures 4C, D**). In the WT and *SFU1* complemented groups, microbes aggregated and surrounded within a matrix of EPS, forming a complex and dense biofilm structure. The *sfu1/sfu1* mutant group showed decreased amount of both microbial density and EPS production, and exhibited loose structure.

To understand the bacterial response, we analyzed the gene expressions related to the glycolysis and EPS metabolism of *S. mutans*. As shown in **Figures 4E**, the *sfu1/sfu1* mutant group showed significantly reduced expression levels of EPS synthesis genes *gtfB*/*C* and increased expression levels of EPS decomposition genes *dexA*/*B*, while no significant difference was found in glucan-binding protein genes *gbpB*/*C*.

## Discussion

Various studies have suggested the correlation between *C. albicans* and dental caries (Kashyap et al., 2024; Lu et al., 2023; Francis et al., 2025; Man et al., 2025). Investigation on the cariogenic mechanism of *C. albicans* is of great significance in clinical prevention and treatment. This study demonstrates the pivotal role played by *SFU1*, the core enzyme for iron-sulfur (Fe-S) cluster biogenesis, in regulating the cariogenic virulence related traits of *C. albicans*, both in monoculture and within cross-kingdom biofilm with *S. mutans*. Our findings reveal that *SFU1* is not only critical for hyphal development, redox homeostasis, and biofilm formation of *C. albicans*, but also dramatically reshapes its cross-kingdom interaction with the caries pathogen *S. mutans*, ultimately altering the cariogenic properties of the biofilm community.

We first investigate the role of *SFU1* in the growth and virulence of *C. albicans* itself. While *SFU1* deletion did not affect the overall growth rate or acid production in planktonic culture, it significantly impaired filamentation on hypha-inducing media, and caused reduced metabolic activity and mitochondrial dysfunction, which were consistent with the central role of Fe-S clusters in the mitochondrial electron transport chain and various metabolic enzymes (Lill and Freibert, 2020). The impaired filamentation directly translated to defective biofilm architecture. The *sfu1/sfu1* mutant exhibited reduced biofilm biomass and downregulation of key hypha-specific (*EFG1*, *UME6*) and adhesin (*ALS3*) genes, suggesting that *SFU1*-mediated Fe-S cluster biogenesis is integral to the regulatory network governing the yeast-to-hypha transition and subsequent biofilm maturation (Lopes and Lionakis, 2022; Noble et al., 2017).

The defects observed in monospecies biofilms were amplified in the dual-species context. Compared to the WT, *SFU1*-deficient *C. albicans* showed a diminished capacity to construct the three-dimensional architectural scaffold of the biofilm. It primarily persisted in the yeast form, which resulted in loose and unstructured aggregates with poor integration of *S. mutans*.

The structural collapse had functional consequences on key cariogenic virulence factors such as lactic acid and EPS production. Lactic acid produced by *S. mutans* through sugar metabolism can serve as the carbon source for *C. albicans* growth (Kim et al., 2017). It is revealed in this study that *SFU1* may be involved in regulating the glycolysis of *S. mutans* and affecting its lactate metabolism by regulating LDH activity, thereby affecting the cariogenic toxicity of the biofilm. These effects may be associated with interspecies signaling and microenvironment modulation, and further experiments are required to clarify the specific mechanisms involved.

Furthermore, the production of EPS was markedly reduced, and the paucity of both biomass and EPS matrix was visually confirmed by CLSM. As the main component of extracellular matrix, EPS can protect, nourish, and enhance adhesion of microorganisms enclosed in it, and promote local pH reduction to form an acidic microenvironment (Lin et al., 2021; Koo et al., 2013). Glucosyltransferases encoded by *gtfB*/*C*/*D* are key factors in EPS synthesis, promoting microbial adhesion and aggregation (Zhang et al., 2021; Wang et al., 2020). The dextran enzyme encoded by *dexA*/*B* can hydrolyze WSG to provide a sugar source for microorganisms (Yan et al., 2023). The glucan binding proteins encoded by *gbpB* and *gbpC* play important roles in the adhesion, colonization, and biofilm formation of *S. mutans* (Lynch et al., 2013; Mieher et al., 2018). In this study, when co-cultured with the *sfu1/sfu1* mutant, *S. mutans* showed downregulated expression of *gtfB/C* and upregulated expression of *dexA/B*, suggesting that the altered fungal phenotype shifts the metabolism of *S. mutans* towards a less matrix-productive state. No significant difference was found in the expression of *gbpB*/*C*, indicating that the loose biofilm structure may resulted in the reduction of microbial biomass and the hyphae of *C. albicans*, rather than regulating adhesion-related genes.

In conclusion, *SFU1* in *C. albicans* emerges as a key regulator of its virulence within a cross-kingdom cariogenic biofilm. The absence of *SFU1* leads to impaired hyphae formation, mitochondrial dysfunction, and oxidative stress in *C. albicans*, which collectively disrupt its ability to form a robust symbiotic partnership with *S. mutans* and distort the gene expression and metabolic behavior of the bacterial partner. Our findings highlight that targeting factors involved in fundamental cellular processes like Fe-S cluster biogenesis in *C. albicans* could be a promising strategy to disrupt the pathogenic synergy in dental caries, offering a novel perspective for anti-biofilm therapies.

## Data Availability

The original contributions presented in the study are included in the article/**Supplementary Material**. Further inquiries can be directed to the corresponding author.
