# Supplementary material for: Candida albicans metabolic adaptation gene SFU1 regulates dual-species biofilm with Streptococcus mutans
Source: Front Cell Infect Microbiol. 2026 Mar 9;16:1795742. doi: 10.3389/fcimb.2026.1795742 (PMC13006602; doi:10.3389/fcimb.2026.1795742)
Supplement: Supplementary file 1 [file Table1.docx]

**Table S1. Primers used in this study.**

| **Name** | **Sequence (5’ to 3’)** | **Purpose** |
| --- | --- | --- |
| *SFU1* 5' flank top + Sal I site  *SFU1* 5' flank bottom + BamH I site  *SFU1* 3' flank top + Xho I site  *SFU1* 3' flank bottom + Bgl II and Sal I sites | ATATACGTCGACAGTACCAGTTACTGTTTGAGAG  ATATACGGATCCCTCTATCCATTTAACAACTTCC  ATATACCTCGAGATGCAAAACAATCCCCTTTC  ATATACAGATCTATGTCGACTCAACTGGACTTGACTCAGAATC | For construction of *SFU1* reconstituted strain |
| ALS1-F  ALS1-R  ALS3-F  ALS3-R  ALS5-F  ALS5-R  EFG1-F  EFG1-R  UME6-F  UME6-R  ACT1-F  ACT1-R | TTCTCATGAATCAGCATCCACAA  CAGAATTTTCACCCATACTTGGTTTC  CAACTTGGGTT A TTGAAACAAAAACA  AGAAACAGAAACCCAAGAACAACCT  TGGCACCACAATGTGAAAAC  CCGGCAGGAACATTTTGATA  TCCAACCACCAGGAATCAGACC  TACCACGTGTCATTTGGGCCAC  TCTTACCTCAATCAGCATTA  CAGCACTAACACTGACACC  TCAGACCAGCTGATTTAGGTTTG  GTGAACAATGGATGGACCAG | For RT-qPCR  (Biofilm formation related genes of *C. albicans*) |
| *C. albicans*-Alexa Fluor 647 | ACCAGACTTGCCCTCC | For species-specific fluorescence label |
| *S. mutans*-Alexa Fluor 488 | ACTCCAGACTTTCCTGAC |  |
| gtfB-F  gtfB-R  gtfC-F  gtfC-R  gtfD-F  gtfD-R  dexA-F  dexA-R  dexB-F  dexB-R  gbpB-F  gbpB-R  gbpC-F  gbpC-R  gyrA-F  gyrA -R | ACACTTTCGGGTGGCTTG  GCTTAGATGTCACTTCGGTTG  CCAAAATGGTATTATGGCTGTCG  TGAGTCTCTATCAAAGTAACGCAG  AATGAAATTCGCAGCGGACTTGAG  TTAGCCTGACGCATGTCTTCATTGTA  AGGGCTGACTGCTTCTGGAGT  AGTGCCAAGACTGACGCTTTG  ATCTGGGGAAATACGGGCAA  TGTTCTGGCATTATCACGGC  AGCAACAGAAGCACAACCATCAG  CCACCATTACCCCAGTAGTTTCC  CCAACAACTCCTGATGAACCAACG  AGCAGCCCCAGTATGTGGAAG  ATTGTTGCTCGGGCTCTTCCAG  ATGCGGCTTGTCAGGAGTAACC | For RT-qPCR  (EPS metabolism related genes of *S. mutans*) |
